# A prognostic model composed of four long noncoding RNAs predicts the overall survival of Asian patients with hepatocellular carcinoma

**DOI:** 10.1002/cam4.3275

**Published:** 2020-07-06

**Authors:** Xuefeng Gu, Hongbo Li, Ling Sha, Wei Zhao

**Affiliations:** ^1^ Medical School Southeast University Nanjing Jiangsu China; ^2^ Department of Liver Disease The Second Hospital of Nanjing Medical School Southeast University Nanjing Jiangsu China; ^3^ Department of Hepatology Infectious Diseases Hospital Affiliated with Soochow University Suzhou Jiangsu China; ^4^ Department of Neurology Affiliated Drum Tower Hospital of Nanjing University Medical School Nanjing China

**Keywords:** ceRNA, hepatocellular carcinoma, lncRNA, overall survival, risk score, TCGA

## Abstract

Based on accumulating evidence, long noncoding RNAs (lncRNAs) are potential biomarkers and therapeutic targets for many diseases, including tumors. In this study, we consulted The Cancer Genome Atlas (TCGA) database to explore the functions and modulatory mechanisms of lncRNAs as competing endogenous RNAs (ceRNAs) in hepatocellular carcinoma (HCC) in Asian patients and constructed a risk scoring system composed of four lncRNAs (SNHG1, STEAP3‐AS1, RUSC1‐AS1, and SNHG3) to predict the outcomes of Asian patients with HCC. The prognostic value of this risk model was validated in the internal validation cohort (n = 157). The stratified survival analysis revealed good performance for the risk model in stratifying clinical features. According to the Cox proportional hazard regression analysis, the four‐lncRNA risk model is an independent prognostic model for Asian patients with HCC. Finally, we developed a nomogram that integrates prognostic signals and other clinical information to predict 1‐, 3‐, and 5‐year overall survival rates. In conclusion, the prognostic lncRNAs identified in our study exerted potential biological effects on the development of HCC. The risk scoring model based on four lncRNAs may be an effective classification tool for assessing the prognosis of Asian patients with HCC.

## Graphical abstract

We constructed a lncRNA‐miRNA‐mRNA competing endogenous RNA (ceRNA) network associated with hepatocellular carcinoma (HCC) in Asia. Based on the ceRNA network, we constructed and assessed a four‐lncRNA model for predicting the survival time of Asian HCC patients.

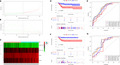

## 1 INTRODUCTION

Hepatocellular carcinoma (HCC) is the sixth most common human cancer worldwide and the fourth leading cause of cancer‐related mortality. In 2018, over 800 000 new HCC cases occurred worldwide and approximately 780 000 patients died due to HCC.^1^ HCC is treated with chemotherapy, radiotherapy, ablation, surgical resection, and liver transplantation^2^, ^3^; however, the overall treatment efficacy is poor. In the United States, the 5‐year survival rate is less than 20%,^4^ while the rate is approximately 5% in developing countries.^5^ According to epidemiological studies, a clear geographical manifestation of HCC is observed based on an increased incidence of hepatitis B virus (HBV) infections and aflatoxin contamination. HCC is most prevalent in East Asia and Southeast Asia,^1^ where approximately 50% of the total global cases and deaths occur in China.^6^ Therefore, in‐depth studies of the molecular mechanisms of HCC in Asian patients must be conducted to screen for new prognostic biomarkers and formulate corresponding therapeutic strategies.

Long noncoding RNAs (lncRNAs) are gradually becoming a hot spot in cancer research. The lncRNA transcripts are more than 200 nucleotides in length, and lncRNAs are known to interact with microRNAs (miRNAs) as competing endogenous RNAs (ceRNAs) to modulate the expression of target genes.^6^, ^7^ Gu et al.^8^ constructed a three‐lncRNA risk scoring system (LOC101927051, LINC00667, and NSUN5P2) to predict the prognosis of small hepatocellular carcinoma. Based on the results of the Kaplan‐Meier analysis, these lncRNAs exhibit significant prognostic value in Asian patients with HCC who do not have a family history of HCC or a history of drinking. Zhang et al.^9^ reported elevated expression of the lncRNA plasmacytoma variant translocation 1 (PVT1) in Asians. Zheng et al.^10^ enrolled 81 patients with HCC and observed a significant decrease in the expression of the long intergenic non‐protein coding RNA 1018 (SRHC) in tumor tissues compared with corresponding noncancerous tissues. Moreover, a critical correlation was observed between the expression of lncRNA SRHC and hepatocyte nuclear factor 4A (HNF‐4A) in HCC, and SRHC may be a key downstream target of HNF‐4A. However, few studies have examined the HCC‐related ceRNA network in Asians, and no risk prognosis model of lncRNAs based on the ceRNA network is available.

In this study, we identified differentially expressed RNAs (DERNAs) in 157 HCC tissues and six nontumor liver tissues from Asian patients in The Cancer Genome Atlas (TCGA) database and constructed a lncRNA‐miRNA‐mRNA ceRNA network related to Asian patients with HCC that included 18 lncRNAs, three miRNAs, and 76 mRNAs. We subsequently established a four‐lncRNA model that predicts survival based on the ceRNA network including these 18 lncRNAs and applied these four lncRNAs as candidate biomarkers for Asian patients with HCC.

## 2 MATERIALS AND METHODS

### 2.1 Data collection

Raw sequencing data for mRNA and miRNA expression profiles associated with HCC were downloaded from the TCGA database (https://portal.gdc.cancer.gov/) using the gdc‐client tool, and complete clinical data from the corresponding patients were subsequently collected from the cBioPortal database (http: //www.cbioportal.org/). After excluding non‐Asian patients and patients with no survival data, mRNA‐seq and lncRNA‐seq data from 157 HCC specimens and six matching normal liver tissue specimens (from 157 Asian patients with HCC) and miRNA‐seq data from 160 HCC specimens and six matching normal liver tissue specimens (from 160 Asian patients with HCC) were acquired. The TCGA clinical data showed that none of the patients underwent adjuvant/postoperative radiation therapy or adjuvant pharmaceutical treatment for the tumors submitted to the TCGA database. Four patients underwent ablation embolization as an adjuvant treatment. Based on the data on the surgical procedures used to determine the definitive diagnosis of the tumors submitted to the TCGA database, only one patient underwent total hepatectomy with transplantation. The differential expression levels of lncRNAs, mRNAs, and miRNAs were further analyzed using edgeR (version 3.22.5) included in the R software package (version 3.6.0). A log2‐fold change >1 and a *P* value <.05 were used as cutoff values for RNA expression. This study fully complied with the publication requirements and guidelines of TCGA. Approval from the ethics committee was unnecessary.

### 2.2 Construction of the ceRNA network

We first predicted and screened the miRNAs that matched the differentially expressed lncRNAs using the miRcode database (http://www.mircode.org/), and the matched miRNA sequences were then modified with the StarBase database (http://starbase.sysu.edu.cn/). Subsequently, the targeted mRNAs were searched using the Perl program based on the miRDB (http://www.mirdb.org/), TargetScan (http://www.targetscan.org/), and miRTarBase (http://mirtarbase.mbc.nctu.edu.tw/php/index.php) databases. The final target genes were selected by consulting all three databases. The differentially expressed mRNAs and the final target genes were cross‐extracted using the VennDiagram R package (version 1.6.20) to obtain differentially expressed mRNAs and construct the ceRNA network. The ceRNA network was created and visualized using Cytoscape v3.7.1.

### 2.3 Functional enrichment analysis

A gene ontology (GO) analysis was conducted on overlapping differentially expressed mRNAs using the bioinformatics database and the Database for Annotation, Visualization, and Integrated Discovery (DAVID) (https://david-d.ncifcrf.gov/). Then, a Kyoto Encyclopedia of Genes and Genomes (KEGG) functional enrichment analysis was performed using the clusterprofiler package of the R software.^11^ A false discovery rate (FDR) <0.05 was used as a screening criterion. The results of the GO and KEGG analyses were displayed using the GOplot package in R software.

### 2.4 Construction of the lncRNA prognostic risk scoring system

A survival analysis based on a univariate Cox proportion model in the R language was used to screen for lncRNAs in the overlapping differentially expressed long noncoding RNAs (DElncRNAs). The screening condition is that the P values were less than 0.05. The Akaike information criterion (AIC) was employed to select the most appropriate prognostic model.^12^ A multivariate Cox proportional hazards regression analysis was then utilized to further identify the candidate genes. Using the median value of the risk score as the cutoff point, patients were divided into low‐risk and high‐risk groups. The Kaplan‐Meier method and the log‐rank test were used to describe the overall survival (OS) of Asian patients with HCC. The accuracy of the prediction generated by the prognostic risk model was analyzed by constructing time‐dependent receiver operating characteristic (ROC) curves.

### 2.5 Verification of the survival predicted by the lncRNA model in the internal validation cohort

We further validated the ability of the prognostic risk model to predict the survival of the internal validation cohort. We used the bootstrap resampling method to create the validation dataset by retrieving data from 157 patients with HCC. This method was recommended for the internal validation of prognostic models.^13^, ^14^ Using the same model as constructed above, the median risk score categorized patients in the validation cohort into low‐risk and high‐risk groups. Subsequently, the Kaplan‐Meier survival curve and time‐dependent ROC curves were plotted to assess the predictive effectiveness of the prognostic model.

### 2.6 Analysis of the effectiveness of the prognostic risk model in clinical subgroups of Asian patients with HCC

The prognostic value of the prognostic risk model was assessed in subgroups with different clinical characteristics, including the Eastern Cooperative Oncology Group (ECOG) score, hepatitis B, alcoholic consumption, histological grade, and tumor node metastasis (TNM) stage. Kaplan‐Meier survival curves and the log‐rank test were performed for the assessment.

### 2.7 Independent analysis of the predictions generated with the prognostic risk model

We comprehensively handled the missing clinical data using the R package “rpart”^15^, ^16^ and By using STATA software version 12.0 (College Station, TX, USA), Univariate and multivariate Cox regression analysis was used to evaluate whether the predictive potential of the prognostic model can be used as an independent prognostic factor for Asian liver cancer patients. The forest plot was created with GraphPad Prism 6.0 software (GraphPad Software, La Jolla, CA, USA).

### 2.8 Construction of nomograms for determining the prognosis of Asian patients with HCC

As nomograms are widely used to predict the outcomes of patients with cancer, we combined all independent prognostic factors to construct nomograms designed to assess the 1‐year, 3‐year, and 5‐year OS rates of Asian patients with HCC. We built the nomogram using the rms package of R. The performance of the nomogram and the predicted and actual probability of survival were further explored using the concordance index (C‐index) and the calibration curves.

## 3 RESULTS

### 3.1 Differentially expressed lncRNAs, mRNAs, and miRNAs

**FIGURE 1:**
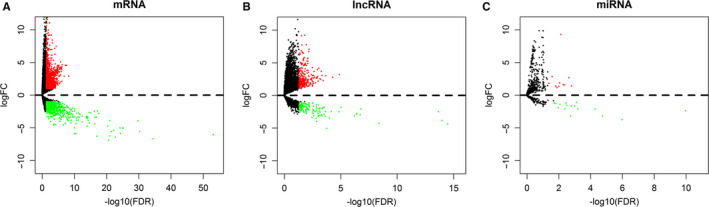
Specific lncRNA‐related ceRNA network and characteristics of the constituent lncRNAs in Asian patients with HCC. The volcano plot shows the expression profiles of mRNAs (A), lncRNAs (B), and miRNAs (C). Red dots indicate upregulated RNAs, and green dots indicate downregulated RNAs. ceRNA: competing endogenous RNA, HCC: hepatocellular carcinoma, lncRNA: long noncoding RNA, miRNA: microRNA

**FIGURE 2:**
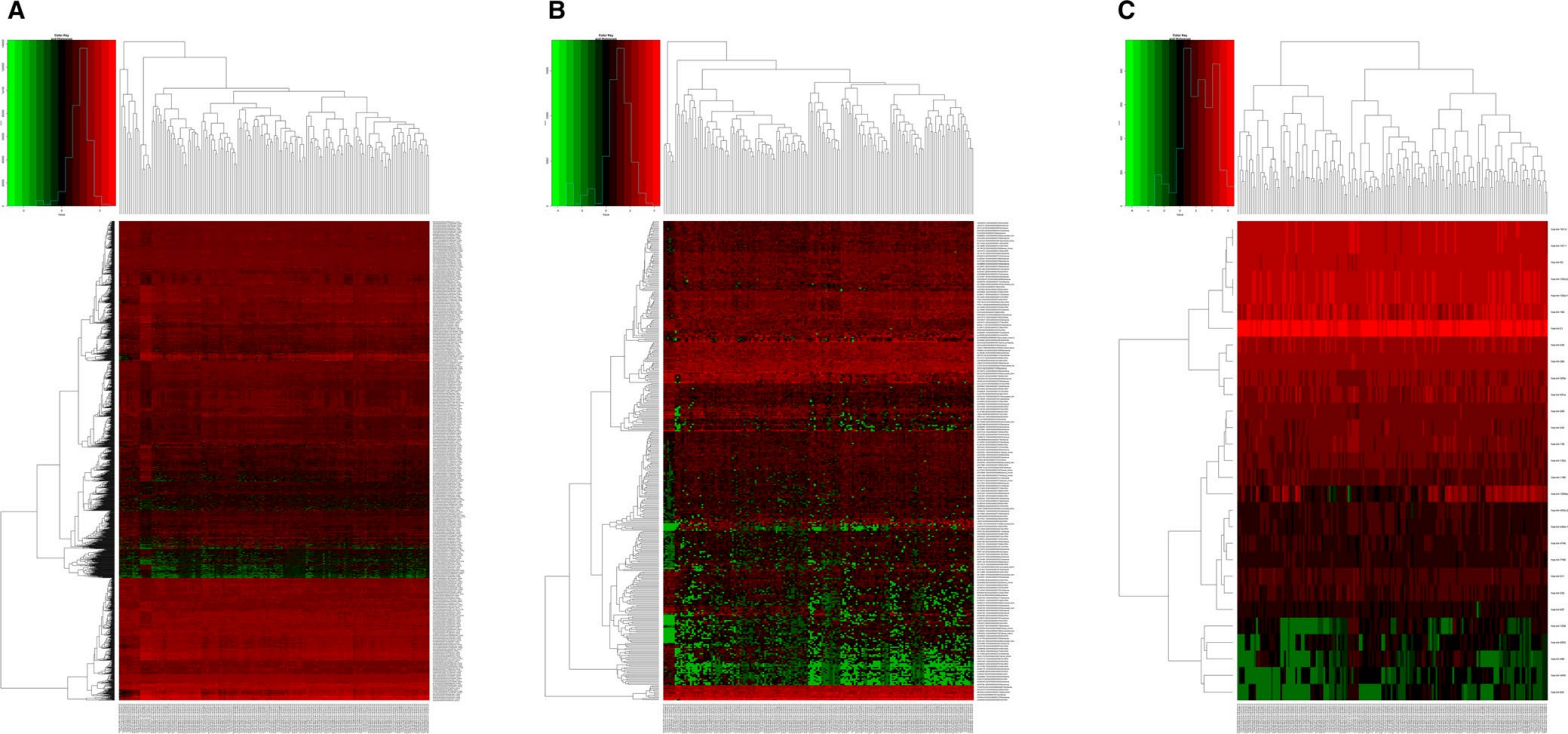
Heatmaps of differentially expressed RNAs in different samples: A, mRNAs, B, lncRNAs, and C, miRNAs. The y‐axis represents RNAs and the x‐axis represents patient samples; red denotes upregulation and green denotes downregulation

We identified 2174 mRNAs (1237 upregulated and 937 downregulated), 376 lncRNAs (249 upregulated and 127 downregulated), and 29 miRNAs (12 upregulated and 17 downregulated) that were differentially expressed between HCC tissues and noncancerous tissues. We generated volcano plots of the expression profiles of mRNAs, lncRNAs, and miRNAs (Figure 1A‐C). Heatmaps of the mRNAs, lncRNAs, and miRNAs showed that the samples with similar characteristics tended to be clustered according to their expression levels (Figure 2A‐C).

### 3.2 The lncRNA‐miRNA‐mRNA ceRNA network

**FIGURE 3:**
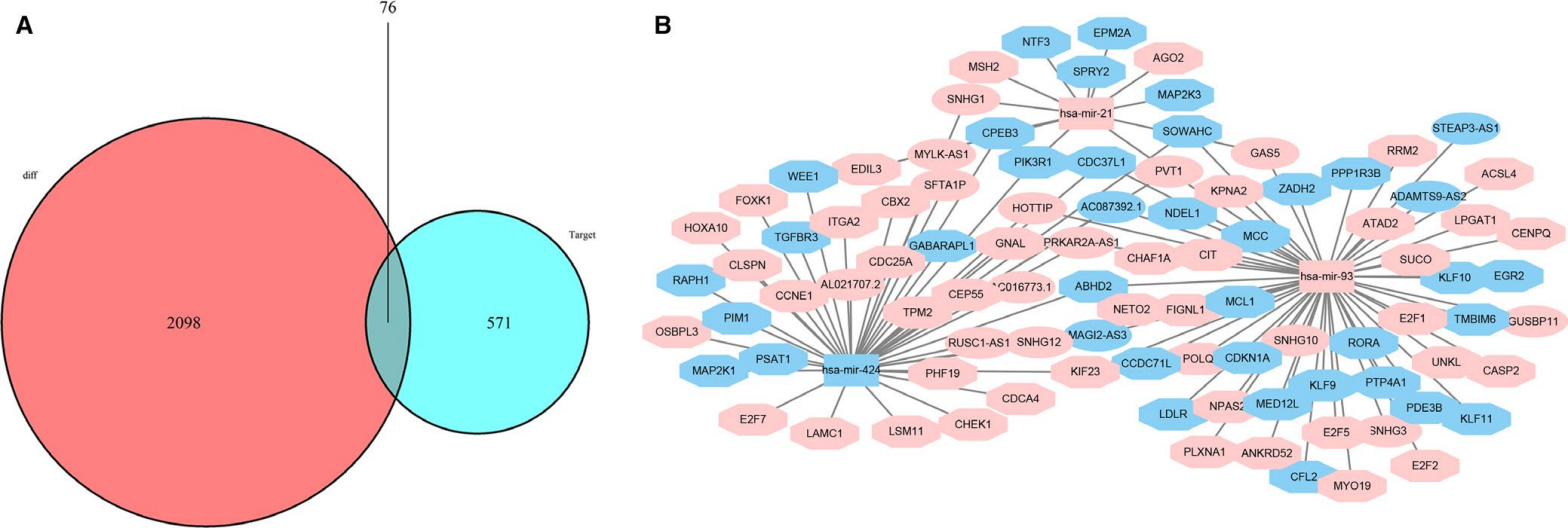
The ceRNA network in Asian patients with HCC. A, Venn diagram of DEmRNAs involved in the ceRNA network. B, The ceRNA network of lncRNAs‐miRNAs‐mRNAs involved in HCC. Ellipses represent lncRNAs, rectangles represent miRNAs, and octagons represent mRNAs. The nodes highlighted in red and blue indicate up‐ and downregulation, respectively. DEmRNAs: differentially expressed mRNAs

We evaluated the lncRNA‐miRNA interactions using miRcode; predicted the target mRNAs of the miRNAs using miRTarBase, miRDB, and TargetScan; and cross‐extracted the selected genes to build the ceRNA network. Finally, 76 differentially expressed mRNAs (DEmRNAs) were included in the ceRNA network (Figure 3A). In summary, we screened over 18 lncRNA nodes, 3 miRNA nodes, and 76 mRNA nodes to construct the differential expression profile for the ceRNA network. The ceRNA network was visualized using Cytoscape (Figure 3B).

### 3.3 Functional analysis

**FIGURE 4:**
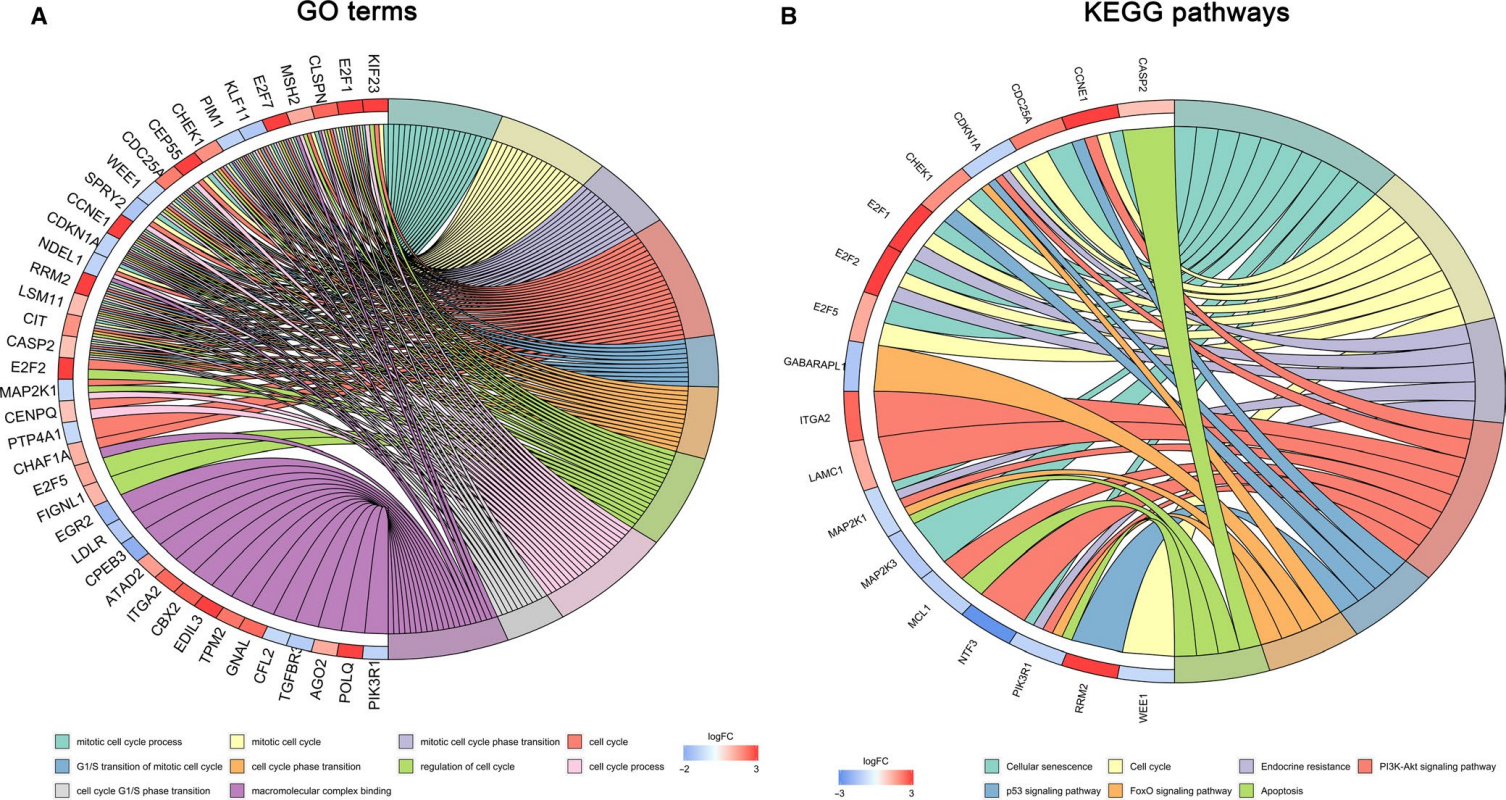
Enrichment analysis of DEmRNAs involved in the ceRNA network. A, The top 10 significantly enriched pathways identified in the DEmRNA GO enrichment analysis. B, Significantly enriched KEGG pathways of DERNAs (FDR <0.05). DEmRNA: differentially expressed mRNA, GO: gene ontology, KEGG: Kyoto Encyclopedia of Genes and Genomes, FDR: false discovery rate.

A biological enrichment analysis was conducted using DAVID GO terms and the clusterprofiler package of R to investigate the biological functions of the components of the ceRNA network. Overlapping DEmRNAs were significantly enriched in 12 GO bioprocess categories (FDR < 0.05). The top five GO terms were “mitotic cell cycle process,” “mitotic cell cycle,” “mitotic cell cycle phase transition,” “cell cycle,” and “G1/S transition of the mitotic cell cycle” (Figure 4A). The significantly enriched KEGG pathways were “cellular senescence,” “cell cycle,” “PI3K‐Akt signaling pathway,” “endocrine resistance,” “p53 signaling pathway,” “FoxO signaling pathway,” and “apoptosis” (Figure 4B).

### 3.4 Construction and verification of the prognostic risk model

**FIGURE 5:**
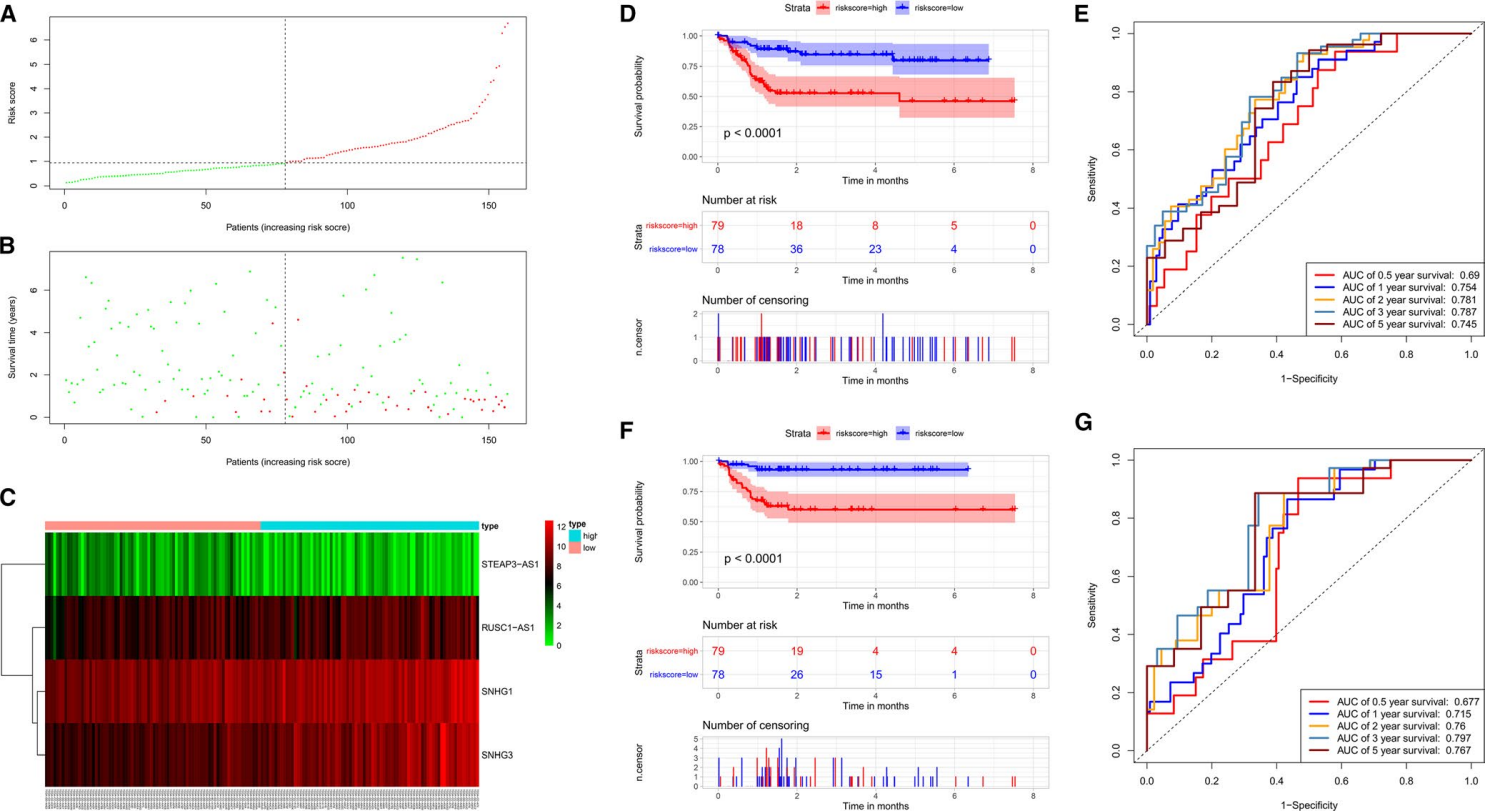
Construction of a four‐lncRNA risk model for Asian patients with HCC. A, Distribution of lncRNA risk scores for 157 Asian patients with HCC. B, Survival status of each patient. C, Heatmap showing the expression of the four lncRNAs corresponding to each of the above samples. Red: high expression; green: low expression. D, Kaplan‐Meier analysis of the OS of Asian patients with HCC using the risk scores based on the four characteristic lncRNAs. E, Analysis of the predicted survival based on the time‐dependent ROC curve for the prognostic model. F, Analysis of the Kaplan‐Meier curve for the four‐lncRNA prognostic model risk scores in the internal validation cohort to validate the OS of Asian patients with HCC. G, Analysis of time‐dependent ROC curves for survival predicted by the prognostic model and verified in the internal validation cohort. OS: overall survival, ROC: receiver operating characteristic.

We completed the univariate and multivariate Cox proportional hazards regression analyses to assess the correlation between lncRNA expression levels and patient survival. Four lncRNAs, namely, SNHG1, STEAP3‐AS1, RUSC1‐AS1, and SNHG3, were screened from 18 DElncRNAs, and a four‐lncRNA prediction model was created (Figure 5A‐C). The risk scoring system of the prediction model was established as follows: risk score = (0.3768 * SNHG1 expression level) + (−0.2702 * STEAP3‐AS1 expression level) + (0.2998 * RUSC1‐AS1 expression level) + (0.2666 * SNHG3 expression level). We then calculated the risk score for each patient and performed a survival analysis using the Kaplan‐Meier method and the log‐rank test. Patients with high‐risk scores had a worse OS than patients with low‐risk scores (log‐rank *P* < .001, Figure 5D). The prognostic power of the four‐lncRNA prediction model was assessed by calculating the area under the curve (AUC) of the time‐dependent ROC curve; a higher AUC indicated better performance of the model. The AUCs for the 0.5‐, 1‐, 2‐, 3‐, and 5‐year survival of the four lncRNA biomarkers were 0.690, 0.754, 0.781, 0.787, and 0.745, respectively, indicating that the prediction model exhibited good sensitivity and specificity (Figure 5E).

The model was subsequently tested in the internal validation cohort to assess the ability of the four‐lncRNA risk scoring system to predict the survival of Asian patients with HCC. No significant differences in basic characteristics were observed between the internal validation cohort and model group (Table 1). The 157 patients in the internal validation cohort were divided into a low‐risk group (n = 78) and a high‐risk group (n = 79). The survival of the patients in the low‐risk group was remarkably longer than patients in the high‐risk group (Figure 5F, *P* < .001). The 0.5‐, 1‐, 2‐, 3‐, and 5‐year AUCs of the time‐dependent ROC curves were 0.677, 0.715, 0.760, 0.797, and 0.767, respectively (Figure 5G).

**TABLE 1:** The clinical features of patients with hepatocellular carcinoma in the model cohort and validation cohort

Characteristics	Model group (n = 157)	Validation cohort (n = 157)	*P* value
Death [n (%)]	44 (28.03%)	33 (21.02%)	.192
Survival time (mean ± SD, mo)	25.547 ± 22.745	23.164 ± 19.218	.926
Age (mean ± SD, y)	55.166 ± 11.554	54.108 ± 10.509	.290
Gender (male/female)	123/34	122/35	.892
BMI (≥25/<25/NA kg/m2)	33/120/4	31/120/6	.794
Hepatitis B (Yes/No/NA)	88/57/12	98/49/10	.516
Alcohol consumption (Yes/No/NA)	44/101/12	54/93/10	.465
AFP (≤ 20/>20/NA)	61/60/36	67/63/27	.440
Vascular invasion (Yes/No/NA)	46/83/28	43/94/20	.347
Histological grade (G4/G3/G2/G1)	11/68/64/14	12/74/56/15	.834
AJCC TNM stage (IV/III/II/I/NA)	1/41/34/79/2	2/40/30/81/4	.864
Residual tumor (R0/R1R2RX/NA)	145/3/9	142/4/11	.829
ECOG (4/3/2/1/0/NA)	2/12/14/25/81/23	4/11/7/21/98/16	.282

Note

The Mann‐Whitney *U* test or *t* test was performed to compare the differences in continuous variables, as appropriate. Fisher’s exact test or the Chi‐square test was performed to compare the differences in categorical variables, as appropriate.

Abbreviations: SD, standard deviation, BMI, body mass index, NA, missing data, AFP, Alpha‐fetoprotein, AJCC, American Joint Committee on Cancer, TNM, tumor node metastasis, ECOG, Eastern Cooperative Oncology Group.

### 3.5 Correlation between the risk score and clinical features of Asian patients with HCC

We analyzed the relationship between various clinical features and our risk score in Asian patients with HCC (Table 2). A high‐risk score correlated with alpha‐fetoprotein (AFP) level (*P* = .001), the histological grade (*P* = .046), and ECOG score (*P* = .001) of Asian patients with HCC. In contrast, high‐risk scores were not correlated with gender, age, alcohol consumption, soluble hepatitis B antigen (HBsAg) level, body mass index (BMI), vascular invasion, and TNM stage (all *P* > .05).

**TABLE 2:** Correlation between the clinicopathological variables and four‐lncRNA risk score in the model cohort.

Parameters	Groups	N	Four‐lncRNA risk score				χ^2^	*P* value
			High‐risk	%	Low‐risk	%		
Age (years)	≥60	57	23	29.11	34	43.59	3.557	.059
	<60	100	56	70.89	44	56.41		
Gender	Female	34	20	25.32	14	17.95	1.256	.262
	Male	123	59	74.68	64	82.05		
BMI (kg/m^2^)	≥25	33	17	22.08	16	21.05	0.024	.877
	<25	120	60	77.92	60	78.95		
Alcohol consumption	No	101	50	68.49	51	70.83	0.094	.759
	Yes	44	23	31.51	21	29.17		
Hepatitis B	No	57	32	43.84	25	34.72	1.262	.261
	Yes	88	41	56.16	47	65.28		
AFP (ng/ml)	≤20	61	19	33.93	42	64.62	11.332	.001*
	>20	60	37	66.07	23	35.38		
Vascular invasion	No	83	35	57.38	48	70.59	2.446	.118
	Yes	46	26	42.62	20	29.41		
Histologic grade	Grade 1+2	78	33	41.77	45	57.69	3.979	.046*
	Grade 3+4	79	46	58.23	33	42.31		
TNM stage	Stage I+II	113	55	70.51	58	75.32	0.454	.500
	Stage III+IV	42	23	29.49	19	24.68		
ECOG	0	81	32	47.06	49	74.24	10.352	.001*
	>0	53	36	52.94	17	25.76		

* *P* < .05. The Chi‐square test or Fisher’s exact test was performed to compare the differences in categorical variables, as appropriate.

### 3.6 Survival rate predicted by the prognostic risk model for clinical subgroups of Asian patients with HCC

**FIGURE 6:**
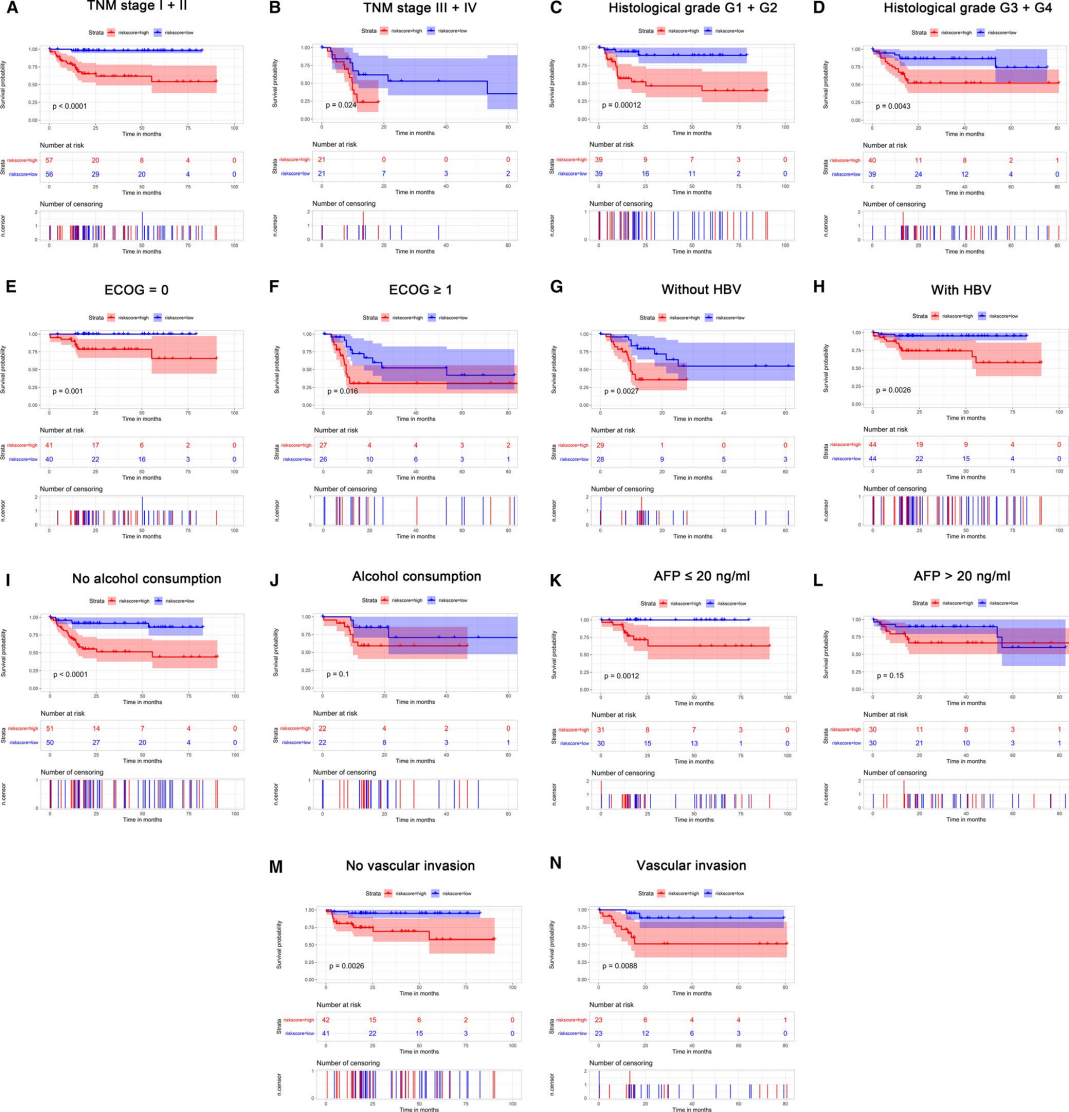
Confirmation and development of the lncRNA risk scoring system using a TCGA cohort. Analysis of Kaplan‐Meier curves for OS values of the subgroups of Asian patients with HCC using the four‐lncRNA risk model, including (A) TNM I + II phase, (B) TNM III + IV phase, (C) histology G1 + G2 phase, (D) histology G3 + G4 phase, (E) ECOG = 0, (F) ECOG ≥ 1, (G) without HBV, (H) with HBV, (I) alcohol consumption (no) , (K) AFP≤ 20 ng/mL, (M) no vascular invasion, and (N) vascular invasion. No significant difference in Asian patients with a history of alcohol consumption (J) or an AFP> 20 ng/ml (L) (all P > 0.05). P values were determined using the log‐rank test. TCGA: The Cancer Genome Atlas, R: residual tumor, ECOG: Eastern Cooperative Oncology Group, HBV: hepatitis B virus, AFP: Alpha‐fetoprotein

The potential of the four‐lncRNA prognosis risk model was further analyzed in the TCGA cohort. The patients in each clinical subgroup were divided into low‐risk and high‐risk groups based on the cutoff value. The risk scoring system was used to perform a prognostic analysis in the subgroups stratified by ECOG scores, HBV, alcohol consumption, AFP level, vascular invasion, histological grade, and TNM stage. The high‐risk groups in the prognosis risk assessment of the subgroups of TNM stage I + II, TNM stage III + IV, histological grade of G1 + G2, histological grade of G3 + G4, ECOG = 0, ECOG ≥1, without HBV, with HBV, no vascular invasion, and vascular invasion exhibited a poor OS (Figure 6A‐H, M and N). The prognostic risk model was more suitable for Asian patients without a history of alcohol consumption (Figure 6I, *P* < .001) or an AFP ≤20 ng/mL (Figure 6K, *P* = .001), but it did not provide a significant result for Asian patients with a history of alcohol consumption (Figure 6J, *P* = .1) or an AFP >20 ng/ml (Figure 6L, *P* = .15).

### 3.7 OS prediction for Asian patients with HCC

**FIGURE 7:**
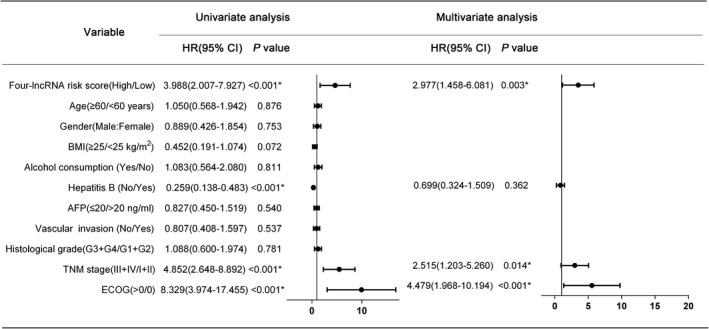
Univariate and multivariate Cox regression analyses of Asian patients with HCC

The risk score of the four‐lncRNA prognosis model, hepatitis B, TNM stage, and ECOG score displayed prognostic value in determining the OS in the univariate Cox regression analysis (*P* < .05). The multivariate Cox regression analysis further revealed that the TNM stage (hazard ratio (HR) = 2.515, *P* = .014), ECOG score (HR = 4.479, *P* < .001), and the risk score of the four‐lncRNA model (HR = 2.977, *P* = .003) were independent prognostic factors associated with OS (Figure 7).

**FIGURE 8:**
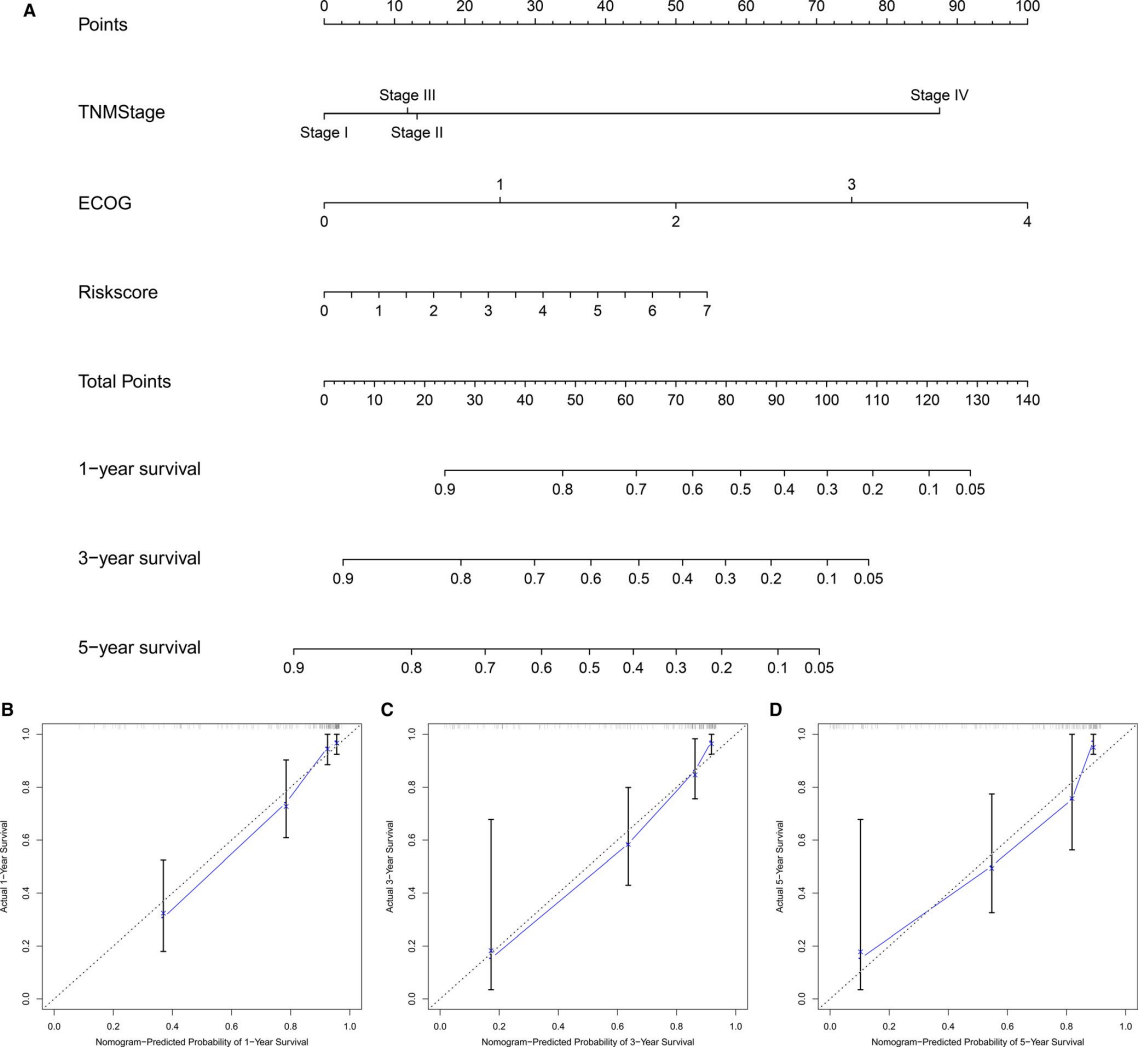
Establishment of a nomogram for predicting the OS of Asian patients with HCC. (A) Nomogram used to predict the 1‐, 3‐, and 5‐year survival rates of Asian patients with HCC. Total points were calculated by adding the corresponding points for each individual covariate included in the scale. Then, 1‐, 3‐, and 5‐year survival rates were obtained by directly converting the total points. Calibration chart of the nomogram used to predict the (C) 1‐, (D) 3‐, and (E) 5‐year OS rates. The predicted probability and actual probability of OS are plotted on the x‐ and y‐axes, respectively

**FIGURE 9:**
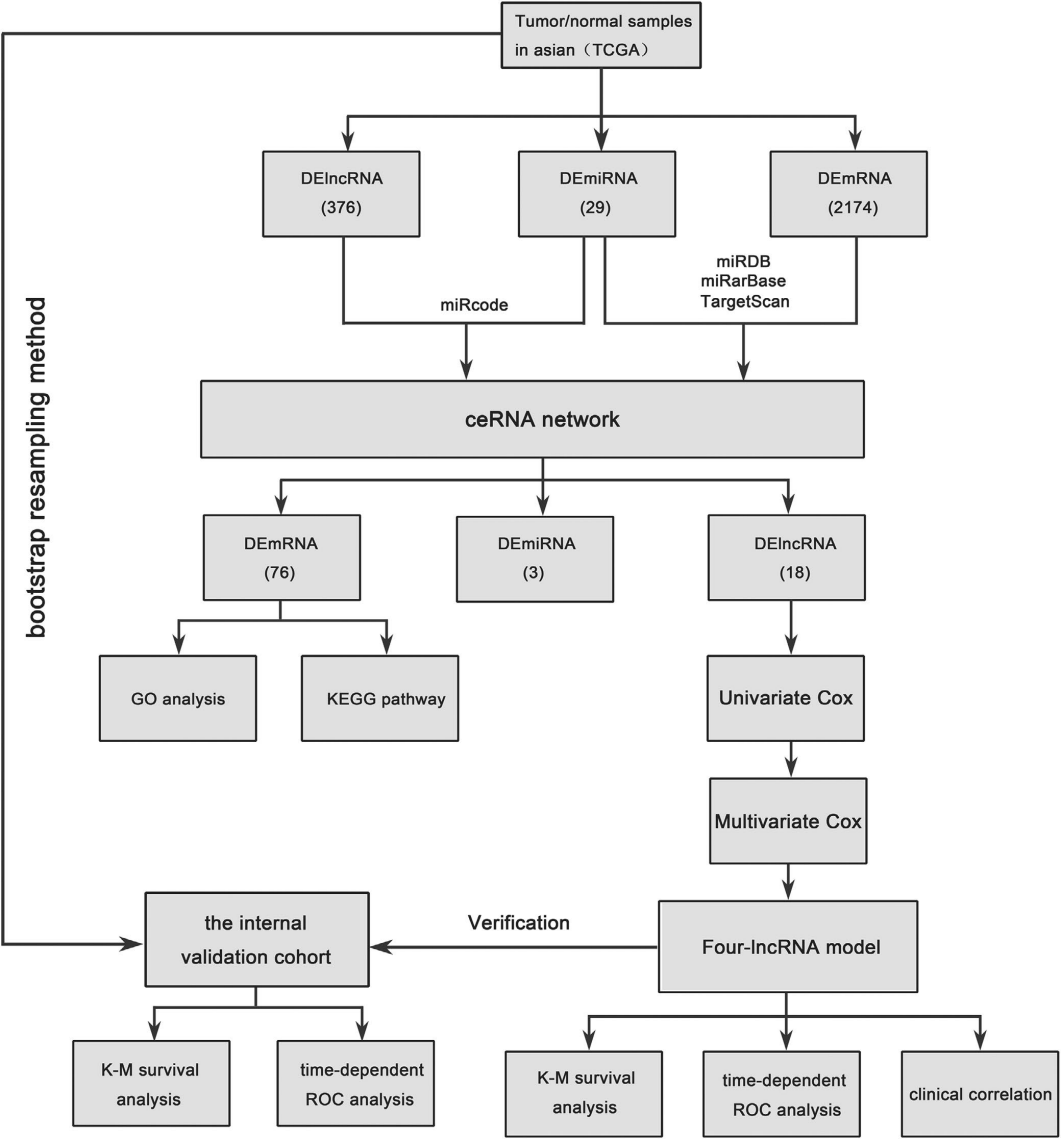
Flow chart of the bioinformatics analysis

A nomogram that integrated the risk score of the four‐lncRNA model and clinical pathological features was developed to predict survival probabilities in Asian patients with HCC. The predicted 1‐year, 3‐year, and 5‐year OS rates are shown in the nomogram (Figure 8A). The C‐index for the OS prediction was 0.828 (*P* < .001). The nomogram calibration curves displayed good agreement between the predicted 1‐year, 3‐year, and 5‐year OS rates and actual observations (Figure 8B‐D). Figure 9 shows a flow diagram of the bioinformatics analysis.

## 4 DISCUSSIONS

HCC is a serious global health issue, particularly in Asia. Due to its complex molecular and cellular heterogeneity, its incidence is increasing annually.^17^ Therefore, reliable biomarkers and genetic signatures for use as therapeutic targets and prognosis prediction factors are very important for HCC. In recent years, a growing number of studies have reported important roles for noncoding RNAs in various biological processes, such as epigenetic regulation, chromosomal remodeling, transcriptional regulation, and posttranslational modification.^18^ Furthermore, the abnormal expression of lncRNAs can disrupt important biological processes, including cell proliferation, migration, and invasion, and lead to malignant transformation and even tumorigenesis.^7^ Although lncRNAs exert substantial effects on HCC development, the impacts of lncRNAs on HCC have not been completely elucidated. In the present study, we initially reported the construction of a ceRNA network for Asian patients with HCC patients, and a four‐lncRNA prognostic risk model was successfully established based on the relationship between the lncRNA expression profile in the ceRNA network and clinical outcomes of Asian patients with HCC.

Subsequently, time‐dependent ROC and Kaplan‐Meier survival curves confirmed that the four‐lncRNA signature had a robust prognostic value for predicting survival. A multivariate Cox regression analysis also confirmed that the four‐lncRNA signature was an independent prognostic indicator for Asian patients with HCC.

Regarding the characteristics of the four lncRNAs, STEAP3‐AS1 was identified as a protective factor (HR < 1), while the other three lncRNAs (SNHG1, RUSC1‐AS1, and SNHG3) were identified as hazard factors (HR > 1). Among these lncRNAs, Yan et al.^19^ developed a 16‐lncRNA prognostic model in the ceRNA network constructed for HCC and found that SNHG1 was also an important lncRNA involved in the construction of the ceRNA network and prognostic model, consistent with our results. SNHG1 is significantly upregulated in HCC,^20^, ^21^, ^22^ and SNHG1 directly inhibits the expression of miR‐195 and promotes the development and progression of HCC.^23^ SNHG1 also promotes HCC cell proliferation by inhibiting the expression of p53 and the p53‐target genes Bcl2‐associated X (BAX), FAS, and cyclin‐dependent kinase inhibitor 1A (CDKN1A).^24^ The overexpression of SNHG1 attenuates sorafenib‐induced apoptosis and autophagy in sorafenib‐resistant HCC cells by activating the Akt pathway through SLC3A2.^25^ SNHG3 expression is crucially elevated in HCC tissues compared with corresponding noncancerous tissues,^22^, ^26^ and SNHG3 expression strongly correlates with the malignant behaviors and unfavorable prognosis of patients with HCC.^26^ Moreover, SNHG3 also induces the epithelial‐mesenchymal transition of HCC cells by activating the miR‐128/CD151 cascade.^27^ The detailed functions of two new lncRNAs (STEAP3‐AS1 and RUSC1‐AS1) in HCC have not been previously studied and deserve further exploration.

Finally, ECOG score, TNM stage, and the four‐lncRNA signature were included in the nomogram to achieve better prognostic prediction power, which is more conducive to evaluations by physicians. If the nomogram is applied in clinical practice in the future, patients at a high risk of tumor‐related death according to the prognostic features or nomogram can be identified before surgery, enabling the more timely administration of an appropriate adjuvant therapy and monitoring before and after surgery. However, the risk model must first be confirmed in a larger population‐based sample, and the molecular functions and potential mechanisms of the four independent lncRNAs in HCC require further exploration.

## CONFLICT OF INTEREST

The authors have declared that no competing interest exists.

## AUTHOR CONTRIBUTIONS

WZ designed the study; XFG and HBL collected data; XFG and LS completed the statistics; XFG drafted the manuscript; and WZ supervised the study. All authors read and approved the final manuscript.

## Data Availability

The datasets used and/or analyzed during the current study are available from the corresponding author on reasonable request.
